# Elevated growth temperature decreases levels of the PEX5 peroxisome-targeting signal receptor and ameliorates defects of *Arabidopsis* mutants with an impaired PEX4 ubiquitin-conjugating enzyme

**DOI:** 10.1186/s12870-015-0605-3

**Published:** 2015-09-16

**Authors:** Yun-Ting Kao, Bonnie Bartel

**Affiliations:** Biochemistry and Cell Biology Program, Department of BioSciences, Rice University, Houston, TX USA

## Abstract

**Background:**

Peroxisomes house critical metabolic reactions. For example, fatty acid β-oxidation enzymes, which are essential during early seedling development, are peroxisomal. Peroxins (PEX proteins) are needed to bring proteins into peroxisomes. Most matrix proteins are delivered to peroxisomes by PEX5, a receptor that forms transient pores to escort proteins across the peroxisomal membrane. After cargo delivery, a peroxisome-tethered ubiquitin-conjugating enzyme (PEX4) and peroxisomal ubiquitin-protein ligases mono- or polyubiquitinate PEX5 for recycling back to the cytosol or for degradation, respectively. *Arabidopsis pex* mutants β-oxidize fatty acids inefficiently and therefore fail to germinate or grow less vigorously. These defects can be partially alleviated by providing a fixed carbon source, such as sucrose, in the growth medium. Despite extensive characterization of peroxisome biogenesis in *Arabidopsis* grown in non-challenged conditions, the effects of environmental stressors on peroxisome function and *pex* mutant dysfunction are largely unexplored.

**Results:**

We surveyed the impact of growth temperature on a panel of *pex* mutants and found that elevated temperature ameliorated dependence on external sucrose and reduced PEX5 levels in the *pex4-1* mutant. Conversely, growth at low temperature exacerbated *pex4-1* physiological defects and increased PEX5 levels. Overexpressing PEX5 also worsened *pex4-1* defects, implying that PEX5 lingering on the peroxisomal membrane when recycling is impaired impedes peroxisome function. Growth at elevated temperature did not reduce the fraction of membrane-associated PEX5 in *pex4-1*, suggesting that elevated temperature did not restore PEX4 enzymatic function in the mutant. Moreover, preventing autophagy in *pex4-1* did not restore PEX5 levels at high temperature. In contrast, MG132 treatment increased PEX5 levels, implicating the proteasome in degrading PEX5, especially at high temperature.

**Conclusions:**

We conclude that growth at elevated temperature increases proteasomal degradation of PEX5 to reduce overall PEX5 levels and ameliorate *pex4-1* physiological defects. Our results support the hypothesis that efficient retrotranslocation of PEX5 after cargo delivery is needed not only to make PEX5 available for further rounds of cargo delivery, but also to prevent the peroxisome dysfunction that results from PEX5 lingering in the peroxisomal membrane.

**Electronic supplementary material:**

The online version of this article (doi:10.1186/s12870-015-0605-3) contains supplementary material, which is available to authorized users.

## Background

Peroxisomes house important metabolic reactions including β-oxidation. Oilseed plants, like *Arabidopsis thaliana*, β-oxidize fatty acids to provide energy for early seedling development before photosynthesis is established [1]. Because this β-oxidation is peroxisomal, dependence on an external source of fixed carbon, such as sucrose, during germination is a hallmark of peroxisome-defective mutants [2, 3].

Peroxisomes can derive from the endoplasmic reticulum and proliferate by division [4]. Morphology and numbers of peroxisomes can vary depending on the cell type, developmental stage, or environmental conditions [5, 6]. Peroxins (PEX proteins) function in peroxisome biogenesis and/or matrix protein import [4, 7]. Fully folded or oligomerized proteins can be post-translationally imported into the peroxisomal matrix by the peroxisomal import machinery [8]. PEX5 recognizes and delivers proteins carrying peroxisome targeting signal type 1 (PTS1), often a C-terminal tripeptide (*e.g.*, SKL) [9]. The receptor-cargo complexes translocate cargo with the assistance of docking peroxins (PEX13 and PEX14); this importomer forms transient pores on the peroxisomal membrane to deliver cargo into the peroxisome matrix [10, 11].

After cargo delivery, PEX5 is recycled from the membrane back to the cytosol with the assistance of a peroxisome-tethered ubiquitin-conjugating enzyme (PEX4; tethered by PEX22) [12, 13] and RING-finger peroxins (PEX2, PEX10, PEX12) [14, 15]. In yeast, PEX4 and PEX12 monoubiquitinate PEX5 for recycling and further rounds of cargo delivery whereas UBC4 and PEX2 polyubiquitinate PEX5, which targets PEX5 for proteasomal degradation [16]. Ubiquitinated PEX5 is recognized and removed from the peroxisomal membrane by a complex of the PEX1 and PEX6 ATPases [17–19] and PEX26, which recruits the PEX1-PEX6 heterohexamer to the peroxisome [19–21].

Although *Caenorhabditis elegans* and *Drosophila melanogaster* direct essentially all matrix proteins to peroxisomes via the PEX5-PTS1 system [22–24], peroxisomes in various yeasts, plants, and mammals also can import proteins bearing N-terminal PTS2 nonapeptides (R[L/I/Q]X_5_HL). PTS2 proteins are recognized and imported by PEX7 [25, 26]. PEX5 and PEX7 are interdependent in plants [25, 27, 28] and mutually enhance cargo-receptor interactions in mammals [29]. In plants, the protease DEG15 cleaves the N-terminal PTS2 region after delivery to the peroxisome matrix [30, 31]. In mammals, damaged PEX7 can be ubiquitinated and degraded by the proteasome [32], but the mechanism by which undamaged PEX7 is recycled remains unclear.

Ubiquitin modification can target PEX5 for recycling or degradation [16]. Moreover, accumulating evidence suggests that balancing PEX5 targeting and retrotranslocation is important for normal peroxisome function [14, 33]. In this study, we demonstrate that elevated growth temperature reduces PEX5 levels in mutants defective in PEX5 recycling. We implicate proteasomal degradation rather than autophagy in this decrease. We hypothesize that reducing overall PEX5 levels relieves the detrimental effects of membrane-associated PEX5 in *pex4-1* and ameliorates the associated physiological defects.

## Results

### Growth at elevated temperature ameliorates the peroxisomal defects of *pex4-1*

Peroxisomal fatty acid β-oxidation provides fixed carbon and energy to germinating *Arabidopsis* seedlings [1]. Peroxisomal mutants that inefficiently perform β-oxidation fail to germinate or grow less vigorously [2, 3]. These defects can be partially reversed by supplementing the growth medium with a fixed carbon source, such as sucrose, which bypasses the need for β-oxidation. As a result, peroxisomal mutants have shorter hypocotyls or do not germinate without sucrose when grown at normal temperature (22 °C) (Additional file 1A).

**Fig. 1:**
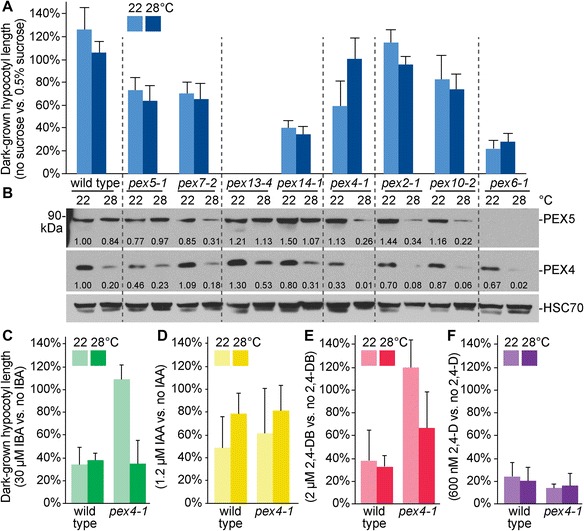
High temperature ameliorates physiological defects and reduces PEX5 levels of *pex4-1.* Physiological consequences of growth temperature on *pex* mutants. Seedlings were grown in the dark at 22 or 28 °C with or without 0.5 % sucrose (**a**), or on media containing 0.5 % sucrose with or without 30 μM IBA (**c**), 1.2 μM IAA (**d**), 2 μM 2,4-DB (**e**), or 600 nM 2,4-D (**f**). Dark-grown hypocotyl lengths were normalized to the corresponding mean of 0.5 % sucrose treatment. Means of normalized dark-grown hypocotyl lengths and standard deviations of the means are shown (*n* ≥ 17 for panel A and *n* ≥ 12 for panels C-F). **b** Protein extracts of dark-grown seedlings from 0.5 % sucrose-supplemented plant nutrient media in panel (**a**) were processed for immunoblotting. The membrane was serially probed with indicated antibodies. HSC70 was used to monitor protein loading. The positions of molecular mass markers (in kDa) are indicated on the left. Band intensities were quantified using ImageJ; levels of PEX5 or PEX4 were normalized to the corresponding HSC70 band prior to normalizing to the 22 °C wild-type band to give the listed numbers

To examine the effect of temperature on mutants with impaired peroxisome function, we surveyed peroxisome-defective mutants for sucrose dependence at normal (22 °C) and elevated (28 °C) growth temperatures. We tested mutants defective in matrix protein receptors (*pex5-1*, *pex7-2*) [3, 27], receptor docking (*pex13-4*, *pex14-1*) [34, 35], and receptor recycling (*pex4-1*, *pex2-1*, *pex10-2*, *pex6-1*) [13, 14, 17]. Growth at 28 °C increased dark-grown hypocotyl lengths (Additional file 1A) but did not markedly alter sucrose dependence in wild type or most peroxisome-defective mutants tested (Fig. 1a, Additional file 1A). Interestingly, we found that high temperature ameliorated the sucrose dependence of dark-grown *pex4-1* seedlings (Fig. 1a). At 22 °C, *pex4-1* hypocotyls were shorter without sucrose supplementation; however, at 28 °C, *pex4-1* hypocotyls were similarly long with or without sucrose (Additional file 1A). This restoration of sucrose independence by growth at high temperature was specific to *pex4-1*; the sucrose dependence of *pex5-1*, *pex7-2*, *pex14-1*, *pex2-1* and *pex10-2* was unchanged or very slightly exacerbated at high temperature, and *pex13-4* did not germinate without sucrose at either temperature (Fig. 1a). We therefore focused on the *pex4-1* mutant to elucidate the molecular changes in peroxisome function that accompany growth at high temperature.

**Fig. 2:**
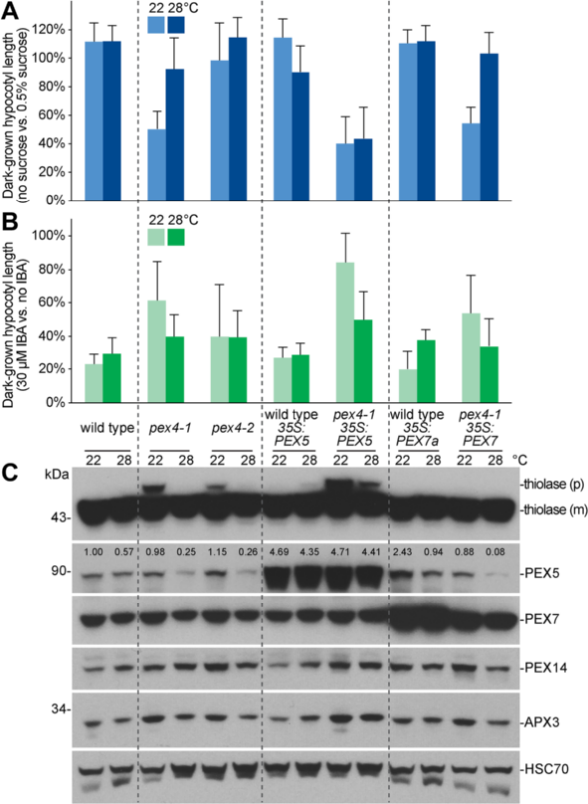
Overexpressing PEX5 but not PEX7 worsens the peroxisomal defects of *pex4-1.* **a**, **b** Seedlings were grown as in the legend of Fig. 1. Means of normalized dark-grown hypocotyl lengths and standard deviations of the means are shown (*n* ≥ 18). *PEX5* was overexpressed in wild type and *pex4-1* using the *35S:PEX5* construct [17]. *PEX7* was overexpressed in wild type using the *35S:PEX7a* construct [27] and in *pex4-1* using the *35S:PEX7* construct [25]. **c** Protein extracts of dark-grown seedlings from 0.5 % sucrose-supplemented plant nutrient media were processed for immunoblotting. The membrane was serially probed with the indicated antibodies. Thiolase is synthesized as a PTS2-containing precursor (p) and cleaved in the peroxisome into a mature (m) form. HSC70 was used to monitor protein loading. The positions of molecular mass markers (in kDa) are indicated on the left. Band intensities were quantified using ImageJ; levels of PEX5 were normalized to the corresponding HSC70 band prior to normalizing to the 22 °C wild-type band to give the listed numbers

In addition to fatty acids, indole-3-butyric acid (IBA) is β-oxidized in peroxisomes to indole-3-acetic acid (IAA), which inhibits cell elongation [3, 36, 37]. Similarly, the synthetic auxin precursor 2,4-dichlorophenoxybutyric acid (2,4-DB) is β-oxidized in peroxisomes to 2,4-dichlorophenoxyacetic acid (2,4-D) [2]. Consequently, wild-type hypocotyls are short following growth on IBA or 2,4-DB whereas peroxisomal mutants often have longer hypocotyls [38] (Additional file 1A, B). Like sucrose dependence (Fig. 1a), we found that growth at high temperature partially restored IBA (Fig. 1c) and 2,4-DB (Fig. 1e) responsiveness to the *pex4-1* mutant. To determine whether this restored IBA responsiveness stemmed from improved peroxisome function or generally increased responsiveness to auxin, we tested the response of *pex4-1* to IAA and 2,4-D, which do not require peroxisomal chain shortening for biological activity. We found that *pex4-1* responded like wild type to both IAA (Fig. 1d) and 2,4-D (Fig. 1f) at both normal and elevated growth temperatures. We concluded that growth at elevated temperature improves peroxisome-related physiology in the *pex4-1* mutant.

The *Arabidopsis pex4-1* mutation alters a conserved proline residue and impairs PEX4 function [13], but the impact of this mutation on PEX4 levels has not been reported. We developed an antibody to *Arabidopsis* PEX4 (Additional file 2) and examined PEX4 levels in our various mutants following growth at normal or elevated temperatures. We detected PEX4 in all of the mutants grown at 22 °C and found PEX4 levels were generally reduced following growth at high temperature (Fig. 1b). In the *pex4-1* mutant grown at 22 °C, pex4-1 protein levels were reduced compared to wild type (Fig. 1b), suggesting that the Pro123Leu mutation destabilizes the pex4-1 protein. pex4-1 levels were further reduced in *pex4-1* grown at 28 °C (Fig. 1b), indicating that high temperature did not ameliorate *pex4-1* physiological defects by restoring pex4-1 protein levels back to wild-type PEX4 levels.

In yeast, the PEX5 PTS1 receptor is retrotranslocated from the peroxisomal membrane by the PEX1-PEX6 ATPase complex following ubiquitination by the PEX2-PEX10-PEX12 ubiquitin-protein ligase complex assisted by the PEX4 ubiquitin-conjugating enzyme [16, 39]. In *Arabidopsis*, mutation of these receptor-recycling peroxins can result in PEX5 destabilization, as in the *pex6-1* mutant [17], or in excessive PEX5 membrane association suggestive of inefficient retrotranslocation, as in the *pex4-1* mutant [33] and a *pex12* mutant [40]. Moreover, *Arabidopsis pex7* mutants display reduced PEX5 levels accompanied by PTS1 import defects in light-grown but not dark-grown seedlings [27]. We used immunoblotting to examine PEX5 levels in our panel of mutants and found that all of the dark-grown mutants except *pex6-1* accumulated detectable levels of PEX5 when grown at 22 °C. However, PEX5 levels were clearly reduced following growth in the dark at high temperature in several mutants (Fig. 1b), especially in *pex7-2* and in the receptor-recycling mutants (*pex4-1*, *pex2-1*, and *pex10-2*). We confirmed that PEX4 is needed to maintain PEX5 levels at elevated growth temperature by using an intronic *pex4* mutant (*pex4-2*). Although *pex4-2* did not display obvious physiological defects (Fig. 2a, b and Additional file 1C), we found similar high temperature-induced PEX5 reduction in both *pex4-1* and *pex4-2* (Fig. 2c).

### Overexpressing PEX5 exacerbates the peroxisomal defects of *pex4-1*

Because PEX5 is inefficiently retrotranslocated from the peroxisomal membrane in *pex4-1* [33], and because both sucrose dependence and PEX5 levels were reduced in *pex4-1* following growth at elevated temperature (Fig. 1a, b), we tested whether overexpressing PEX5 using the constitutive cauliflower mosaic virus (CaMV) *35S* promoter might exacerbate *pex4-1* defects. Whereas overexpressing PEX5 in wild type did not confer IBA resistance or sucrose dependence in dark-grown seedlings (Fig. 2a, b and Additional file 1C), we found that overexpressing PEX5 increased the IBA resistance (Fig. 2b) and heightened the thiolase PTS2 processing defect (Fig. 2c) of dark-grown *pex4-1* seedlings, again suggesting a key role for PEX5 levels or localization in *pex4-1* physiological defects. Growth at elevated temperature only partially ameliorated the increased IBA resistance and PTS2 processing defects of *pex4-1 35S:PEX5* (Fig. 2b, c) and failed to rescue the sucrose dependence of *pex4-1 35S:PEX5* (Fig. 2a). In contrast, overexpressing PEX7, the PTS2 matrix protein receptor, did not worsen *pex4-1* defects but rather appeared to rescue the mild thiolase PTS2-processing defect of *pex4-1* at 22 °C (Fig. 2c). Overexpression of either PEX5 or PEX7 did not markedly alter levels of PEX14 or peroxisomal ascorbate peroxidase (APX3), two peroxisomal membrane proteins (Fig. 2c).

To determine whether PEX5 levels were affected by PEX4 overexpression, we compared PEX5 levels in *pex4-1* seedlings transformed with a genomic copy of *PEX4* or a *PEX4* cDNA driven from the CaMV *35S* promoter [13]. As previously shown [13], both constructs fully rescued the sucrose dependence and IBA resistance of dark-grown *pex4-1* seedlings (Additional file 2). Moreover, the IBA sensitivity, sucrose independence, and PEX5 levels in these lines also resembled wild type following growth at 28 °C, despite the excess PEX4 that accumulated in the *35S:PEX4* line (Additional file 2). These results suggest that PEX4 is not limiting for PEX5 degradation in wild type.

### Physiological and molecular defects of *pex4-1* are enhanced by mutations in *PEX5*

**Fig. 3:**
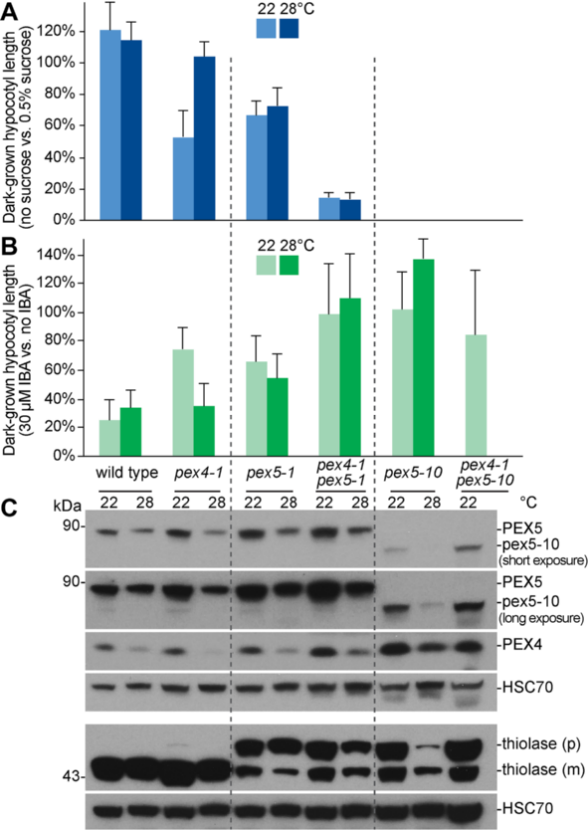
Peroxisomal defects of *pex4-1* are exacerbated by the *pex5-1* and *pex5-10* mutations*.* **a**, **b** Seedlings were grown as in the legend of Fig. 1. Means of normalized dark-grown hypocotyl lengths and standard deviations of the means are shown (*n* ≥ 7). No bars are shown for *pex4-1 pex5-10* at 28 °C because of extremely poor germination rate (one seed germinated on 0.5 % sucrose-supplemented plant nutrient medium out of approximately 100 seeds plated; none germinated without sucrose or with 30 μM IBA). **c** Protein extracts of dark-grown seedlings from 0.5 % sucrose-supplemented plant nutrient media were processed for immunoblotting. The membrane was serially probed with the indicated antibodies. Thiolase is synthesized as a PTS2-containing precursor (p) and cleaved in the peroxisome into a mature (m) form. HSC70 was used to monitor protein loading. The positions of molecular mass markers (in kDa) are indicated on the left

Because overexpressing PEX5 exacerbated *pex4-1* mutant defects and because growth at high temperature ameliorated *pex4-1* mutant defects while reducing PEX5 levels, we assessed how reducing PEX5 function through mutation would affect *pex4-1* mutant defects. Two *Arabidopsis pex5* mutants have been described: the missense *pex5-1* allele [3] specifically disrupts PTS2 import [25] whereas the *pex5-10* T-DNA insertion allele [13] expresses reduced levels of a truncated PEX5 product and disrupts both PTS1 and PTS2 import [27, 41]. We found that the *pex4-1 pex5-1* double mutant was more sucrose dependent (Fig. 3a) and IBA resistant (Fig. 3b) than either single mutant and that these defects were not ameliorated by growth at elevated temperature (Fig. 3a, b). Similarly, combining *pex4-1* with *pex5-10* resulted in seedlings that, like *pex5-10*, remained fully sucrose dependent (Fig. 3a) and IBA resistant (Fig. 3b) and were more impaired than *pex5-10* when grown with sucrose supplementation (Additional file 1D). Moreover, the germination defect of *pex5-10* [41] was exacerbated by *pex4-1*; double mutant seedlings germinated less efficiently than *pex5-10* when incubated at 22 °C and generally failed to germinate when incubated at 28 °C, so we could not assess IBA responsiveness or sucrose dependence of *pex4-1 pex5-10* at elevated temperature.

Immunoblotting revealed that growth at high temperature further reduced levels of the truncated pex5-10 protein but seemed to reduce the thiolase PTS2 processing defect in *pex5-10* (Fig. 3c). Despite the enhanced physiological defects displayed by the double mutants (Fig. 3a and b), combining *pex4-1* with *pex5-1* or *pex5-10* did not dramatically alter the thiolase PTS2 processing defects (Fig. 3c).

### Growth at reduced temperature exacerbates the peroxisomal defects of *pex4-1*

**Fig. 4:**
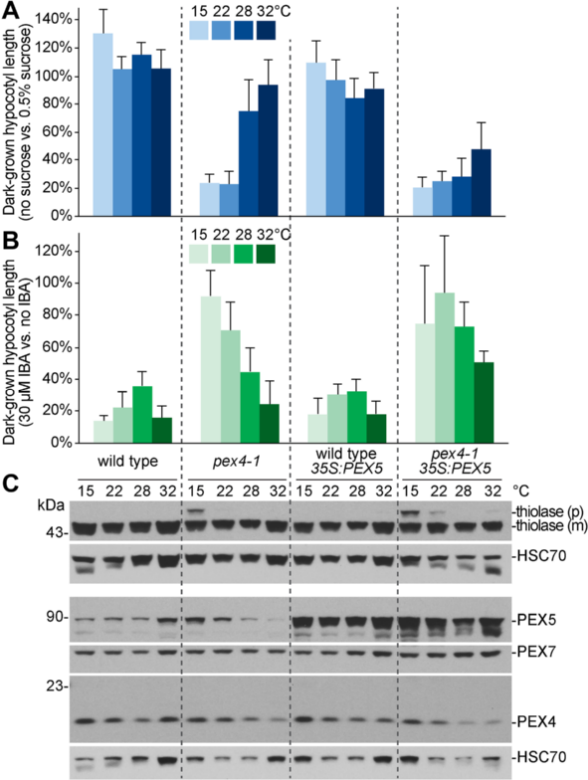
Low temperature worsens the peroxisomal defects of *pex4-1.* Seedlings were grown in the dark at 15, 22, 28, or 32 °C with or without 0.5 % sucrose (**a**) or 30 μM IBA (**b**). Means of normalized dark-grown hypocotyl lengths and standard deviation of the means are shown (*n* ≥ 20). **c** Protein extracts of dark-grown seedlings from 0.5 % sucrose-supplemented plant nutrient media were processed for immunoblotting. The membrane was serially probed with the indicated antibodies. Thiolase is synthesized as a PTS2-containing precursor (p) and cleaved in the peroxisome into a mature (m) form. HSC70 was used to monitor protein loading. The positions of molecular mass markers (in kDa) are indicated on the left

Because high temperature alleviated the *pex4-1* physiological defects, we tested whether reduced growth temperature would intensify these defects. Although growth at 15 °C did not appear to enhance the sucrose dependence of *pex4-1* (Fig. 4a), we found that *pex4-1* seedlings grown at 15 °C were more IBA resistant than seedlings grown at 22 °C (Fig. 4b, Additional file 1E) whereas wild-type seedlings displayed robust IBA responsiveness when grown at 15 °C. Similarly, the PTS2 processing defect of *pex4-1* was more apparent following growth at reduced temperature (Fig. 4c). Overexpressing PEX5 exacerbated *pex4-1* defects and growing at reduced temperature further worsened the thiolase PTS2 processing defects of *pex4-1* (Fig. 4c). Moreover, PEX5 levels, which did not vary markedly in wild-type seedlings grown at different temperatures, showed a clear negative correlation with growth temperature in the *pex4-1* mutant, with more PEX5 protein accumulating at cooler temperatures and less PEX5 accumulating at elevated growth temperatures (Fig. 4c).

### PEX5 is more membrane associated in *pex4-1*; high temperature does not rescue this defect

**Fig. 5:**
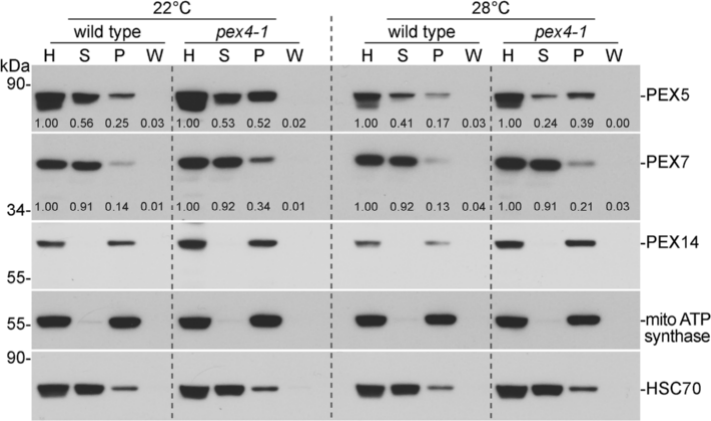
PEX5 is more membrane associated in *pex4-1*; high temperature does not rescue this defect. Extracts from dark-grown seedlings grown at 22 °C or 28 °C were cleared by a low-speed centrifugation to give homogenates (H). After a high-speed centrifugation, supernatants (S) were removed, and pellets were resuspended and spun at high speed to separate the wash supernatants (W) and final pellets (P). Equal volumes of each fraction were processed for immunoblotting by sequential incubation of the membrane with the indicated antibodies. PEX14 and mitochondrial (mito) ATP synthase are integral membrane proteins; HSC70 is mainly cytosolic. The positions of molecular mass markers (in kDa) are indicated on the left. Band intensities were quantified using ImageJ. For each sample, the supernatant, pellet, and wash were normalized to the corresponding homogenate to give the listed numbers

Certain mutants defective in receptor-recycling peroxins, including *pex4-1* and *pex6-1*, display an elevated fraction of membrane-associated PEX5 [33]. We hypothesized that high temperature might increase membrane fluidity and allow more efficient PEX5 retrotranslocation in the *pex4-1* mutant, thus explaining the observed physiological rescue. To test this idea, we used cellular fractionation coupled with immunoblotting to monitor PEX5 localization in seedlings grown at 22 or 28 °C. As expected, the PEX14 membrane peroxin was fully membrane associated in wild type and *pex4-1* at either growth temperature (Fig. 5). As previously observed in light-grown seedlings [33], we found that in extracts from dark-grown wild-type seedlings, PEX5 was mostly soluble following growth at 22 °C and that *pex4-1* had a higher fraction of membrane-associated PEX5 (Fig. 5). We further found that growth at elevated temperature (28 °C) did not notably alter PEX5 membrane association in wild type. Moreover, high temperature did not rescue the high membrane-associated PEX5 defect in *pex4-1*; rather, the PEX5 pellet/supernatant ratio was elevated further when *pex4-1* was grown at high temperature (Fig. 5). We concluded that the physiological rescue observed following growth of *pex4-1* at high temperature did not result from restoration of PEX5 retrotranslocation from the peroxisomal membrane.

PEX7 recognizes and delivers PTS2 cargo, but the PEX7 recycling mechanism is not well understood. Unlike PEX5 levels, PEX7 levels (Fig. 2) or membrane association (Fig. 5) did not noticeably change following growth at elevated temperature.

### High temperature-induced PEX5 reduction in *pex4-1* is not due to autophagy

**Fig. 6:**
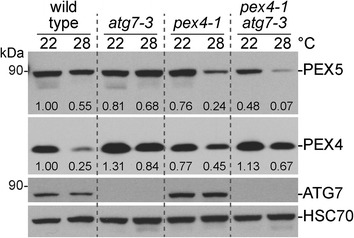
High temperature-induced PEX5 reduction in *pex4-1* does not require autophagy. Plants were grown as in the legend of Fig. 1. Protein extracts of dark-grown seedlings from 0.5 % sucrose-supplemented plant nutrient media were processed for immunoblotting. The membrane was serially probed with the indicated antibodies. HSC70 was used to monitor protein loading. Band intensities were quantified using ImageJ. The levels of PEX5 or PEX4 were normalized to the corresponding HSC70 band prior to normalizing to the 22 °C wild-type band to give the listed numbers

Because growth at elevated temperature reduced overall PEX5 levels in the *pex4-1* mutant, we explored the molecular basis of this reduction. We first tested for the involvement of autophagy in PEX5 degradation. In macroautophagy, an isolation membrane selectively engulfs specific or general cellular components for ultimate degradation in the vacuole [42]. ATG7 is required for lipidation of the ubiquitin-like ATG8 that marks the isolation membrane [42] and is thus required for autophagy of peroxisomes (pexophagy) [43]. We found that preventing autophagy by crossing *pex4-1* to the *atg7-3* null allele [44] did not increase PEX5 accumulation in *pex4-1* at either growth temperature (Fig. 6), suggesting that high temperature-induced PEX5 reduction in *pex4-1* does not require autophagy.

### MG132 treatment implicates ubiquitin-dependent proteasomal degradation in regulating PEX5 levels

**Fig. 7:**
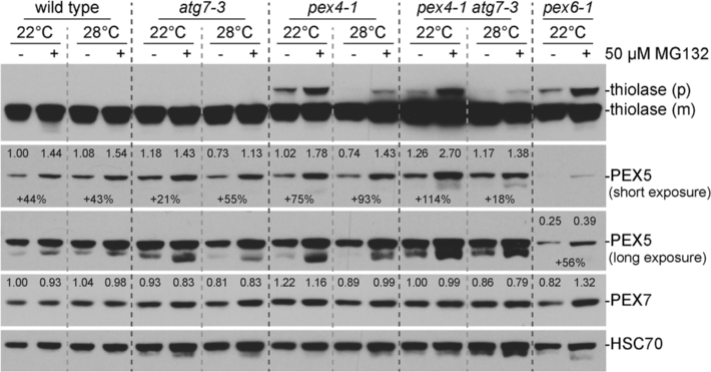
PEX5 degradation is reduced following treatment with the MG132 proteasome inhibitor. One-day stratification and one-day preincubation were performed prior to plating. Plates were placed in yellow light at 22 °C for one day and wrapped in aluminum foil at 22 °C for three days. Seedlings were moved to 0.5 % sucrose-supplemented liquid plant nutrient media with or without 50 μM MG132 in the dark for 24 h at 22 or 28 °C. Protein extracts of dark-grown seedlings were processed for immunoblotting. The membrane was serially probed with the indicated antibodies. Thiolase is synthesized as a PTS2-containing precursor (p) that is cleaved in the peroxisome to the mature (m) form. HSC70 was used to monitor protein loading. Band intensities were quantified using ImageJ. The levels of PEX5 and PEX7 were normalized to the corresponding HSC70 band prior to normalizing to the 22 °C non-MG132-treated wild-type band to give the listed numbers

In yeast, ubiquitinated PEX5 is retrotranslocated from the membrane by the PEX1-PEX6 ATPase complex for recycling, but if this retrotranslocation is slowed, ubiquitination promotes PEX5 degradation by the proteasome [16, 45]. A role for the proteasome in *Arabidopsis* PEX5 degradation has not been directly demonstrated but is implied by the reduced PEX5 levels found in the *Arabidopsis pex6-1* mutant [17]. To test proteasomal involvement in *Arabidopsis* PEX5 degradation, we used a proteasome inhibitor, MG132, to slow ubiquitin-dependent proteasomal degradation [46, 47]. We found that PEX5 levels were similarly elevated following a 24-h MG132 treatment at both normal and elevated temperature in wild type (Fig. 7), suggesting that PEX5 can normally be degraded in a proteasome-dependent manner. PEX5 levels in *pex4-1* grown at either normal or elevated temperature were also increased by MG132 treatment (Fig. 7), suggesting that the proteasome contributes to PEX5 degradation in *pex4-1* as well. Interestingly, the relative increase in PEX5 levels following MG132 treatment was greater in *pex4-1* grown at elevated temperature than in wild type, suggesting that PEX5 degradation by the proteasome is enhanced in *pex4-1* mutants grown at elevated temperature. In contrast to PEX5 levels, the levels of PEX7 were largely unchanged in this experiment (Fig. 7), demonstrating that not all peroxins were similarly sensitive to MG132 treatment.

We also observed that the PTS2-containing precursor of thiolase accumulated to higher levels in *pex4-1* and *pex6-1* mutants following MG132 treatment (Fig. 7). This result suggests that mislocalized cytosolic thiolase can be degraded by the proteasome or that the matrix protein import defects of these mutants were worsened by MG132 treatment.

## Discussion

**Fig. 8:**
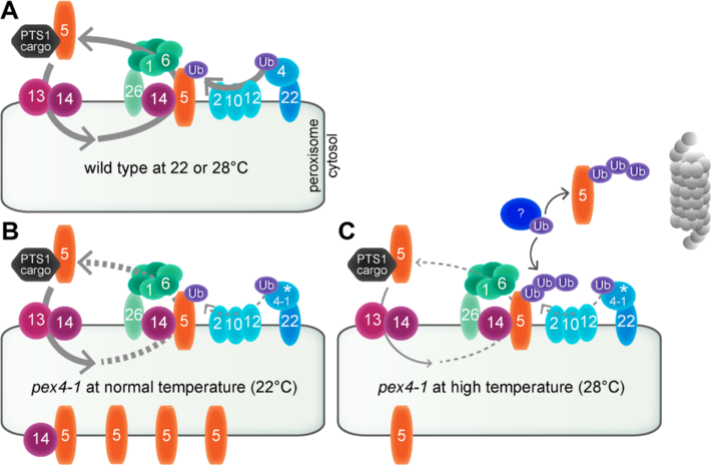
A working model for high-temperature amelioration of *pex4-1* physiological defects. **a** In wild type, PEX5 recognizes and delivers PTS1 cargo into peroxisomes. The peroxisome-tethered ubiquitin conjugating enzyme (PEX4) and RING finger peroxins (PEX2, PEX10, PEX12) collaborate to ubiquitinate PEX5. Ubiquitinated PEX5 is recycled back to the cytosol by the PEX1-PEX6 ATPase complex. **b** At normal growth temperature (22 °C), mutations in *PEX4* slow PEX5 recycling, resulting in elevated membrane-associated PEX5, which contributes to *pex4* physiological defects. These defects are amplified by PEX5 overexpression, implying that these defects do not exclusively result from reduced matrix protein import. **c** At high temperature (28 °C), an unknown ubiquitination enzyme (question mark) promotes ubiquitin-dependent PEX5 degradation, reducing overall PEX5 levels and ameliorating *pex4* defects

PEX4 is a ubiquitin-conjugating enzyme tethered to the peroxisome by PEX22 [13]. PEX4 is necessary for peroxisomal matrix protein import, probably via its role in ubiquitinating the PEX5 matrix protein receptor, which allows efficient retrotranslocation of PEX5 from the membrane by the PEX1-PEX6 ATPase complex (Fig. 8a) [48]. The *Arabidopsis pex4-1* mutant is caused by a Pro123Leu missense mutation and displays a variety of phenotypes suggestive of peroxisome deficiencies, including sucrose dependence, IBA resistance, inefficient PTS2 processing [13], PTS1 import defects [14], and elevated membrane-associated PEX5 [33]. Mounting evidence suggests that PEX5 lingering in the membrane is harmful to peroxisome physiology. For example, overexpressing PEX5 confers sucrose dependence and enhances PTS2 processing defects in *Arabidopsis pex10-2*, a mutant defective in one of the RING-finger peroxins [14]. Furthermore, slightly reducing levels of PEX13, which assists in docking PEX5 at the membrane [35], ameliorates the physiological defects of *pex4-1* [33]. In this work, we found that overexpressing PEX5 exacerbated the sucrose dependence, IBA resistance, and PTS2 processing defects of the *pex4-1* mutant (Figs. 2 and 4, Additional file 1C, E). These findings are consistent with the hypotheses that the PEX4 ubiquitin-conjugating enzyme normally promotes PEX5 retrotranslocation from the peroxisomal membrane and that PEX5 impairs peroxisome physiology if not promptly removed from the membrane after cargo delivery.

Various environmental stimuli can affect peroxisome numbers and functions in plant cells. For example, cadmium and salinity treatments induce production of reactive nitrogen species in peroxisomes [49–51]. Slightly elevated temperature is associated with increased peroxisome numbers in Norway spruce (*Picea abies* L. Karst.) [6] and salt (NaCl) stress promotes peroxisome proliferation in *Arabidopsis* roots [52]. However, the interplay of the environment on peroxisomes in plants with compromised peroxisome function remains largely unexplored. In this study, we found that PEX5 levels are reduced following growth of receptor recycling mutants at elevated temperature (Fig. 1b), suggesting that the activity of the receptor recycling machinery might be temperature-dependent.

We found that growth at elevated temperature rescued the sucrose dependence of dark-grown *pex4-1* seedlings (Fig. 1a) and ameliorated the *pex4-1* thiolase PTS2-processing defect (Figs. 2c and 4c) whereas growth at decreased temperature had opposite effects (Fig. 4). PEX4 levels decreased at high temperature (Figs. 1b, 3, 4c, 6), suggesting that the observed phenotypic rescue was not due to restoration of PEX4 levels in the mutant. Importantly, the elevated ratio of membrane-associated versus cytosolic PEX5 in *pex4-1* was not corrected by growth at high temperature (Fig. 5), indicating that high temperature did not restore pex4-1 enzymatic activity or increase membrane fluidity to facilitate PEX5 retrotranslocation. Reduced overall PEX5 levels accompanied the amelioration of *pex4-1* physiological defects at high temperature (Figs. 1, 2, 3, 4, 6, 7), further supporting the conclusion that membrane-associated PEX5 impairs peroxisome function in *pex4-1*. Blocking autophagy did not prevent the high temperature-induced PEX5 reduction in *pex4-1* (Fig. 6), suggesting that PEX5 was not degraded by autophagy at high temperature in *pex4-1*. In contrast, using MG132 to slow ubiquitin-dependent proteasomal degradation restored PEX5 levels in *pex4-1* grown at high temperature to wild-type levels (Fig. 7). Interestingly, proteasomal degradation also can contribute to heat stress resistance in rice [53]. Together, our data are consistent with a model in which high temperature promotes ubiquitin-dependent proteasomal degradation of PEX5 in *pex4-1*, which reduces membrane-associated PEX5 and relieves *pex4-1* physiological defects (Fig. 8).

In yeast, the PTS1 cargo receptor PEX5, together with the docking peroxin PEX14, forms transient pores in the peroxisomal membrane to deliver cargo into peroxisomes [11]. PEX4 assists the RING-finger peroxins in ubiquitinating PEX5 [54], allowing PEX5 to be returned to the cytosol and thereby removing the membrane pore. How PEX5 topology changes as it cycles between a soluble protein in the cytosol and an integral protein in the peroxisomal membrane is not known. Moreover, it is not clear why overexpressing PEX5 worsens defects in certain peroxisomal mutants (this study and [14]). We speculate that the excess membrane-associated PEX5 in *pex4-1* (this study and [33]) might still be in the pore conformation, altering peroxisome matrix pH, redox status, and/or cofactor availability, thereby disrupting peroxisome metabolism and contributing to the observed physiological defects in *pex4-1*.

Although high-temperature induced reductions in PEX5 levels were associated with ameliorated *pex4-1* peroxisomal defects (Figs. 1, 2, 3, 4), and although PEX5 overexpression (Fig. 2) and low-temperature induced increases in PEX5 levels (Fig. 4) were associated with worsened *pex4-1* defects, reducing PEX5 function by mutation did not reduce *pex4-1* defects. In fact, the *pex4-1 pex5-1* and *pex4-1 pex5-10* double mutants displayed exaggerated peroxisome-related physiological defects compared to the parents (Fig. 3a, b). This enhancement suggests that the pex5-1 and pex5-10 proteins, which do not efficiently import peroxisomal matrix proteins [3, 27, 41], confer additional detriment to peroxisome function when not efficiently retrotranslocated (*i.e.*, in a *pex4-1* mutant). We expect that a *pex5* mutation that reduces PEX5 protein levels without otherwise impairing PEX5 function might ameliorate the peroxisomal defects of *pex4-1*, as suggested by the finding that slightly reducing levels of the PEX13 docking peroxin reduces the physiological defects of *pex4-1* without notably impairing peroxisome function [33].

Yeast PEX14 is required for pexophagy [55], and yeast mutants lacking PEX4 display elevated PEX14 levels [12], consistent with the possibility that PEX4 might directly or indirectly promote pexophagy as well. In mammalian cells, PEX5 brings the tuberous sclerosis complex to the peroxisome, where it regulates the mTORC pathway and autophagy to suppress tumor formation [56]. The WXXX(F/Y) motif in the N-terminal region of PEX5 that binds PEX14 during PTS1 cargo delivery [57–59] is similar to the WXXL ATG8-binding sequence that targets proteins to the autophagy machinery [60]. Although pexophagy recently has been described in *Arabidopsis* [43, 61, 62], the roles of individual plant peroxins in this process have not been reported. We found no evidence that pexophagy was generally induced in *pex4-1*; levels of the peroxisomal membrane proteins PEX14 and APX3 were not reduced in *pex4-1* (Fig. 2c). Interestingly, PEX4 protein was generally less abundant following growth at elevated temperature (Figs. 1b, 3c, 4c, 6), and blocking autophagy moderated this decline and partially restored PEX4 levels in *pex4-1* (Fig. 6). These results suggest that autophagy might contribute to PEX4 degradation at high temperature.

Human peroxisome biogenesis disorders (PBDs) are genetic diseases caused by peroxin mutations and lack effective treatments. PBD patients have developmental delays and neuropathies that often result in early mortality [63]. *Arabidopsis* peroxisomal mutants share some molecular phenotypes with fibroblasts from PBD patients. For example, both human cells with mutations in *PEX6* [64] and *Arabidopsis pex6-1* mutants [17] have low PEX5 levels (*e.g.*, Fig. 1b, 7). Interestingly, low temperature increases membrane-association of PEX5 in normal human cells [64] and increases peroxisomal import in cells from a PBD patient carrying a *PEX6* mutation [65]. It is possible that low temperature slows PEX5 degradation and increases PEX5 levels in *PEX6*-deficient cells. Here, we discovered that growth at high temperature reduced overall PEX5 levels in the *Arabidopsis pex4-1* mutant. Together, these results reinforce the idea that therapies targeting PEX5 levels or localization might improve peroxisome functioning in certain PBD patients.

## Conclusions

We propose a model (Fig. 8) in which PEX5 in the *pex4-1* mutant lingers on the peroxisomal membrane, which impairs peroxisomal functioning. When *pex4-1* seedlings are grown at high temperature, overall PEX5 levels are reduced via an MG132-inhibitable mechanism, presumably proteasomal degradation. This degradation reduces PEX5 accumulation on the peroxisomal membrane. Our results suggest that efficient removal of PEX5 from the peroxisome after cargo delivery is needed not simply to make PEX5 available for further import rounds but also to prevent the peroxisome dysfunction that results from PEX5 lingering in the peroxisomal membrane.

## Methods

### Plant materials and growth conditions

*Arabidopsis thaliana* wild type and mutants were in Columbia-0 (Col-0) background. *atg7-3* [44], *pex2-1* [14], *pex4-1* [13], *pex5-1* [3], *pex5-10* [13], *pex6-1* [17], *pex7-2* [27], *pex10-2* [14], *pex13-4* [35], *pex14-1* [34], Col-0 transformed with *35S:PEX5* [17], Col-0 transformed with *35S:PEX7* [25], Col-0 transformed with *35S:PEX7a* [27], and *pex4-1* transformed with a genomic rescue construct (*PEX4g*) or a *35:PEX4* construct *(PEX4c)* [13] were previously described. *pex4-2* was a gift from Kim Gonzalez and Wendell Fleming.

*pex4-1* was crossed to Col-0 transformed with *35S:PEX5* [17] and to Col-0 transformed with *35S:PEX7* [25]. Lines homozygous for *pex4-1* and the transgene were selected in progeny of the cross by using PCR-based genotyping (Additional file 3). *pex4-1* was crossed to *atg7-3*, *pex5-1*, and *pex5-10*, and the corresponding double mutants were selected from the progeny of the crosses by using PCR-based genotyping (Additional file 3 and [3, 13, 43]).

Surface-sterilized seeds were grown on plant nutrient medium [66] solidified with 0.6 % (w/v) agar and supplemented with the indicated concentrations of sucrose and/or IBA. The IBA stock solution (100 mM) was dissolved in ethanol. One-day stratification in the dark and one-day preincubation in light were performed prior to plating. Plates were placed in yellow light at 22 °C for one day and wrapped in aluminum foil at 22 or 28 °C for four more days, after which dark-grown hypocotyl lengths were measured. For MG132 treatment, plants were grown as described but wrapped in aluminum foil for three days at 22 °C followed by transfer to 0.5 % sucrose-supplemented liquid plant nutrient media with or without 50 μM MG132 for 24 h in the dark at 22 or 28 °C.

### Immunoblotting

A rabbit antiserum was generated against a peptide from near the C-terminus of PEX4 (C126-N141, CDSGNLLRSGDVRGFN) and affinity purified by Bethyl Laboratories (Montgomery, TX). This antibody recognized an ~18 kDa protein in immunoblots of wild-type seedling extracts that was not detected in *pex4-2*, which carries an intronic G-to-A mutation 18 base pairs upstream of the fourth exon of *PEX4*.

Protein extracts from seedlings grown on 0.5 % sucrose-supplemented plant nutrient medium in the dark were processed for immunoblotting. Frozen seedlings were ground with plastic pestles in 1.7 mL tubes, and two volumes of 2X sample buffer (500 mM Tris pH 8.0, 4 % (w/v) lithium dodecyl sulfate, 1 mM EDTA, 20 % (w/v) glycerol, 0.44 mM Coomassie blue G250, 0.332 mM phenol red) were added. Supernatants after centrifugation (4 °C, 13200 rpm, 5 min) were transferred to new tubes, and dithiothreitol (DTT) was added to 50 μM final concentration. Samples were heated at 95 °C for five minutes. Equal volumes of samples, Cruz markers (Santa Cruz Biotech, sc-2035), and pre-stained markers (New England Biolabs, P7708S) were loaded onto NuPAGE or Bolt 10 % (w/v) Bis-Tris gels (Invitrogen) and electrophoresed in 50 mM MOPS free acid, 50 mM Tris base, 0.1 % (w/v) sodium dodecyl sulfate, 1 mM EDTA. Proteins were transferred to Hybond-ECL nitrocellulose membranes (Amersham Hybond ECL, RPN303D) at 24 V for 45 min prior to blocking in 8 % non-fat dry milk in Tris-buffered saline with Tween-20 (20 mM Tris pH 7.5, 150 mM NaCl, 0.1 % (v/v) Tween-20). Membranes were incubated overnight with rabbit primary antibodies diluted in blocking solution that were raised against PEX4 (1:100), PEX5 (1:100, [17]), PEX7 (1:800, [27]), PEX14 (1:10000, Agrisera AS08 372, [67]), thiolase (1:5000, [68]), APX3 (1:1000, [69, 70]), or ATG7 (1:1000, [71]) followed by horseradish peroxidase-linked goat anti-rabbit antibody (1:5000, Santa Cruz Biotechnology sc-2030). A mouse primary antibody against HSC70 (1:50000 – 1:100000, Stressgen HPA-817) paired with horseradish peroxidase-linked goat anti-mouse antibody (1:5000, Santa Cruz Biotechnology sc-2031) was used to monitor protein loading, and mitochondrial ATP synthase (1:2000, Abcam #ab14748, also paired with anti-mouse secondary antibody) was used to validate fractionation experiments. Antibodies were visualized by Western Bright ECL substrate (Advansta K-12045) and exposed on autoradiography film (Genesee 30-810C). Band intensity was analyzed using ImageJ [72].

### Fractionation

Equal weights (750 mg) of 5-day-old dark-grown seedlings were cut with scissors in 1 mL cold fractionation buffer [150 mM pH7.6 Tris, 100 mM sucrose, 10 mM KCl, 1 mM EDTA, 1 mM DTT, 1 mM phenylmethyl sulfonyl fluoride (from a freshly made stock in isopropanol), 16.7 μL plant protease inhibitor cocktail (Sigma P9599), 1 mM N-ethylmaleimide (from a freshly made stock in ethanol)] on ice for five minutes. The chopped tissue and buffer were transferred to a 1 mL Dounce homogenizer (VWR 62400-595), homogenized for 20 strokes, and filtered through Miracloth (Calbiochem). Extracts were cleared by a low-speed centrifugation (50 x g, 10 min) to give homogenates (H). After a high-speed centrifugation (15300 x g, 20 min), the supernatants (S) were removed. The pellets were resuspended and spun at high speed (15300 x g, 20 min) to separate the wash supernatants (W) and final pellets (P). Corresponding volumes of 4X loading buffer were added, and equal volumes of each fraction were used for electrophoresis and immunoblotting with the indicated antibodies.

## Additional files

Additional file 1:
**Dark-grown hypocotyl lengths at different growth temperature.** Physiological consequences of growth temperature on wild type and *pex* mutants (A), wild type and *pex4-1* on various auxins (B), overexpressing PEX5 or PEX7 in wild type or *pex4-1* (C, E), and *pex4-1 pex5* double mutants (D). Seedlings were grown in the dark at indicated temperatures with or without 0.5 % sucrose, 30 μM IBA, 1.2 μM IAA, 2 μM 2,4-DB, or 600 nM 2,4-D. Dark-grown hypocotyl lengths were measured. Means of dark-grown hypocotyl lengths and standard deviations of the means are shown. Normalized data from panels A, B, C, D, and E are presented in Figs. 1a, c-f, 2, 3, and 4, respectively. No bars are shown for *pex4-1 pex5-10* at 28 °C (D) because of the extremely poor germination rate (one seed germinated on 0.5 % sucrose-supplemented plant nutrient medium out of approximately 100 seeds plated; none germinated without sucrose or with 30 μM IBA). (PDF 114 kb) Additional file 2:
**Validation of the PEX4 antibody.** Seedlings were grown as in the legend of Fig. 1. Means of dark-grown hypocotyl lengths (A), normalized dark-grown hypocotyl lengths (B, C), and standard deviations of the means are shown (*n* ≥ 18). (D) Protein extracts of dark-grown seedlings from 0.5 % sucrose-supplemented medium were processed for immunoblotting. The membrane was serially probed with the indicated antibodies. Thiolase is synthesized as a PTS2-containing precursor (p) and cleaved in the peroxisome into a mature (m) form. HSC70 was used to monitor protein loading. The positions of molecular mass markers (in kDa) are indicated on the left. (PDF 3182 kb) Additional file 3:
**PCR-based markers for genotyping mutants.** *pex4-1* carries a C-to-T mutation at the 533^rd^ nucleotide that destroys an *Mnl*Ι restriction enzyme cut site. *pex4-2* carries an intronic G-to-A mutation at the 1085^th^ nucleotide (18 bp upstream of the fourth exon of *PEX4*). We used the dCAPS website (http://helix.wustl.edu/dcaps/dcaps.html, [73]) to design a genotyping marker for *pex4-2. pex5-1* carries a C-to-T mutation at the 2910^th^ nucleotide that destroys an *Eco*RΙ restriction enzyme cut site [3]. *pex5-10* has a T-DNA inserted in exon 5 that results in reduced accumulation of a truncated pex5-10 protein [13]. The *35S:PEX5* construct used a *PEX5* cDNA [17]; for *35S:PEX5* genotyping, *PEX5* primers spanning introns were used to amplify larger genomic *PEX5* or the smaller *PEX5* cDNA products. *PEX7* lacks introns, so a primer from the *35S* promoter and a reverse *PEX7* primer were used to genotype *PEX7-*overexpressing lines. (PDF 71 kb)

## Abbreviations

APX: Ascorbate peroxidase
2,4-D: 2,4-dichlorophenoxyacetic acid
2,4-DB: 2,4-dichlorophenoxybutyric acid
CaMV: Cauliflower mosaic virus
DTT: Dithiothreitol
IAA: Indole-3-acetic acid
IBA: Indole-3-butyric acid
PBD: Peroxisome biogenesis disorder
PEX: Peroxin
PTS: Peroxisome targeting signal
