# Supplementary figures and images for: Elevated growth temperature decreases levels of the PEX5 peroxisome-targeting signal receptor and ameliorates defects of Arabidopsis mutants with an impaired PEX4 ubiquitin-conjugating enzyme
Source: BMC Plant Biol. 2015 Sep 16;15:224. doi: 10.1186/s12870-015-0605-3 (PMC4574000; doi:10.1186/s12870-015-0605-3)

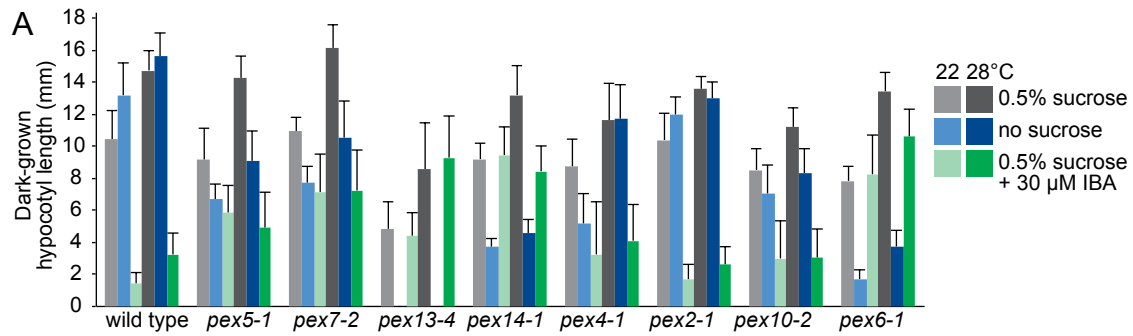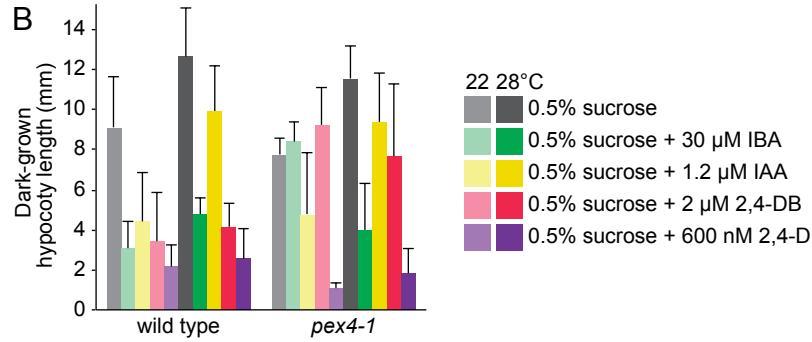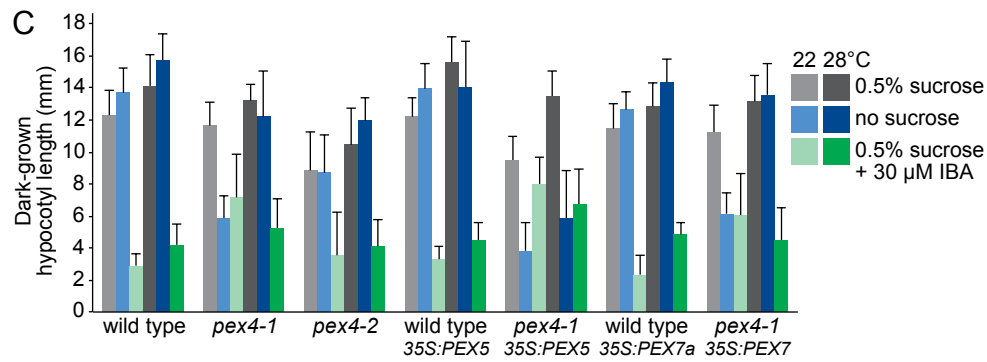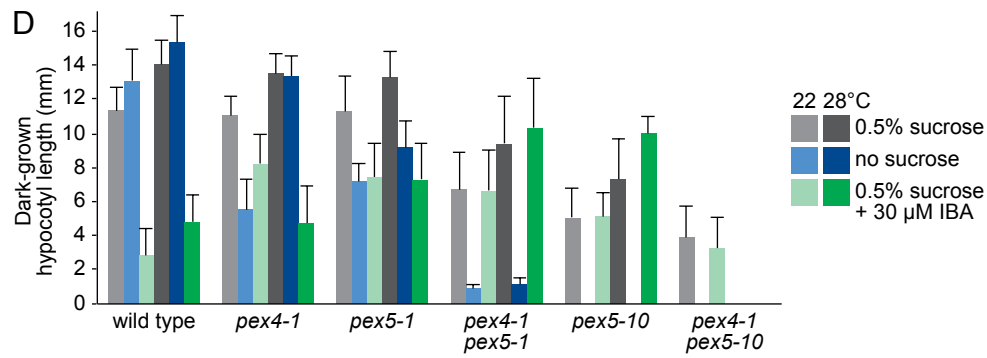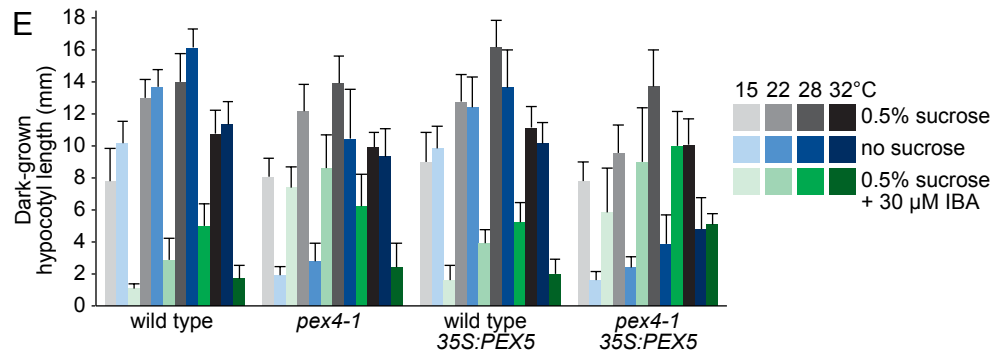

Supplement: Additional file 1: — Dark-grown hypocotyl lengths at different growth temperature. Physiological consequences of growth temperature on wild type and pex mutants (A), wild type and pex4-1 on various auxins (B), overexpressing PEX5 or PEX7 in wild type or pex4-1 (C, E), and pex4-1 pex5 double mutants (D). Seedlings were grown in the dark at indicated temperatures with or without 0.5 % sucrose, 30 μM IBA, 1.2 μM IAA, 2 μM 2,4-DB, or 600 nM 2,4-D. Dark-grown hypocotyl lengths were measured. Means of dark-grown hypocotyl lengths and standard deviations of the means are shown. Normalized data from panels A, B, C, D, and E are presented in Figs. 1a, c-f, 2, 3, and 4, respectively. No bars are shown for pex4-1 pex5-10 at 28 °C (D) because of the extremely poor germination rate (one seed germinated on 0.5 % sucrose-supplemented plant nutrient medium out of approximately 100 seeds plated; none germinated without sucrose or with 30 μM IBA). (PDF 114 kb) [file 12870_2015_605_MOESM1_ESM.pdf]

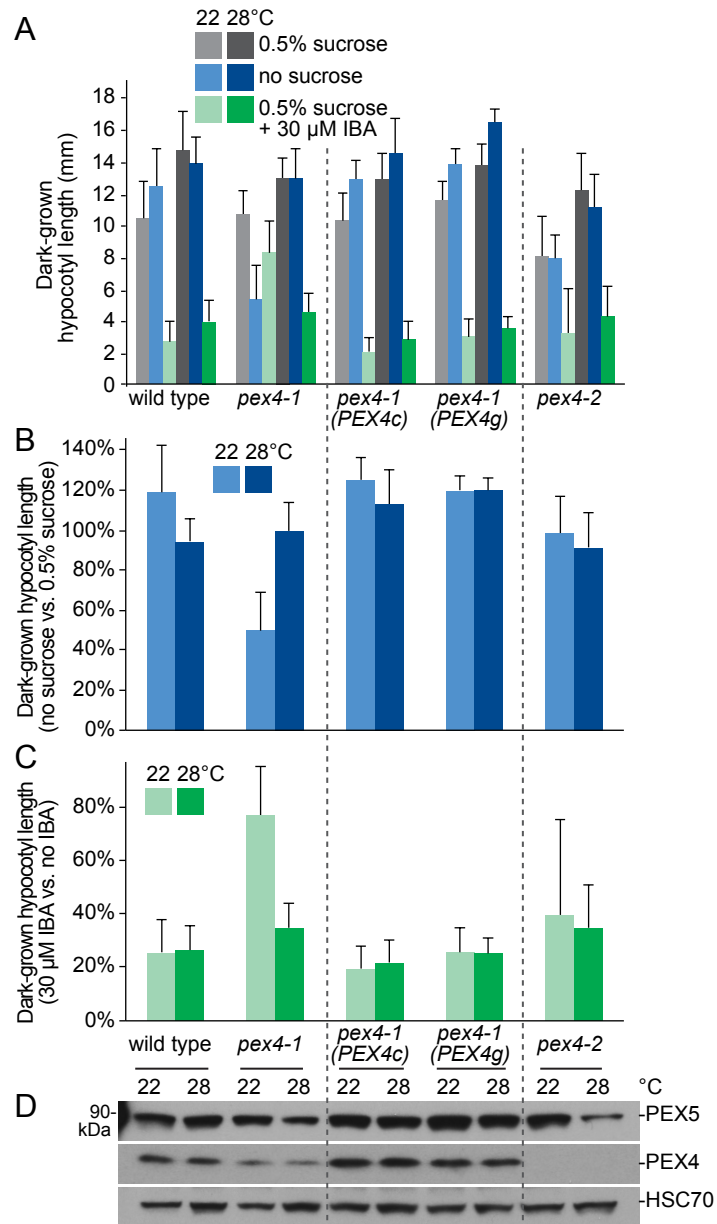

Supplement: Additional file 2: — Validation of the PEX4 antibody. Seedlings were grown as in the legend of Fig. 1. Means of dark-grown hypocotyl lengths (A), normalized dark-grown hypocotyl lengths (B, C), and standard deviations of the means are shown (n ≥ 18). (D) Protein extracts of dark-grown seedlings from 0.5 % sucrose-supplemented medium were processed for immunoblotting. The membrane was serially probed with the indicated antibodies. Thiolase is synthesized as a PTS2-containing precursor (p) and cleaved in the peroxisome into a mature (m) form. HSC70 was used to monitor protein loading. The positions of molecular mass markers (in kDa) are indicated on the left. (PDF 3182 kb) [file 12870_2015_605_MOESM2_ESM.pdf]
