# Supplementary material for: Elevated growth temperature decreases levels of the PEX5 peroxisome-targeting signal receptor and ameliorates defects of Arabidopsis mutants with an impaired PEX4 ubiquitin-conjugating enzyme
Source: BMC Plant Biol. 2015 Sep 16;15:224. doi: 10.1186/s12870-015-0605-3 (PMC4574000; doi:10.1186/s12870-015-0605-3)
Supplement: Additional file 3: — PCR-based markers for genotyping mutants. pex4-1 carries a C-to-T mutation at the 533rd nucleotide that destroys an MnlΙ restriction enzyme cut site. pex4-2 carries an intronic G-to-A mutation at the 1085th nucleotide (18 bp upstream of the fourth exon of PEX4). We used the dCAPS website (http://helix.wustl.edu/dcaps/dcaps.html, [73]) to design a genotyping marker for pex4-2. pex5-1 carries a C-to-T mutation at the 2910th nucleotide that destroys an EcoRΙ restriction enzyme cut site [3]. pex5-10 has a T-DNA inserted in exon 5 that results in reduced accumulation of a truncated pex5-10 protein [13]. The 35S:PEX5 construct used a PEX5 cDNA [17]; for 35S:PEX5 genotyping, PEX5 primers spanning introns were used to amplify larger genomic PEX5 or the smaller PEX5 cDNA products. PEX7 lacks introns, so a primer from the 35S promoter and a reverse PEX7 primer were used to genotype PEX7-overexpressing lines. (PDF 71 kb) [file 12870_2015_605_MOESM3_ESM.pdf]

Additional file 3. PCR-based markers for genotyping mutants.

Genotype	Primers	Restriction enzyme	Product size (bp)	
			Wild type	Mutant
atg7-3	LB1-SAIL (GCCTTTTCAGAAATGGATAAATAGCCTTGCTTCC)<br>ATG7-5 (GTTGCCATGTCTAATCCAGTCAGG)	-	none	350
ATG7	ATG7-5 (GTTGCCATGTCTAATCCAGTCAGG)<br>ATG7-23 (GTTGCCATGTCTAATCCAGTCAGG)	-	281	none
pex4-1	PEX4-A (TGCATCTCTTTTTATAACAACCTTCTCC)<br>PEX4-B (GAACTAGAACCGAACGGGAACCAAACC)	*Mnl*I	201,<br>91	292
pex4-2	PEX4-16 (ATTCGGGTTGCTTCAGTTCG)<br>PEX4-dCAPS-RsaI (CTTAGAAGATTCCCTTTTTTCCATAGCAAG)	*Rsa*I	139	108,<br>31
pex5-1	PEX5-B (TCATCAATAATAAGTTCACCACGGCTCATCT)<br>PEX5-32 (GTTGGACGACATATCTCTTTTCTGG)	*Eco*RI	77,<br>59,117	77,<br>176
pex5-10	LB1-SALK (CAAACCAGCGTGGACCGCTTGCTGCAACTC)<br>PEX5-21 (GATATCAAATGCGACTCAAACACTGATGAC)	-	none	405
PEX5	PEX5-3 (GTCGTTGGCTGAATATTTTGTTCGGC)<br>PEX5-21 (GATATCAAATGCGACTCAAACACTGATGAC)	-	541	none
35S:PEX5	PEX5-38 (TGAAGACCAACAGATAAGG)<br>PEX5-39 (CCCATTGGAGGCATAGG)	-	264	264,<br>168
35S:PEX7	35S-F (GGATGACGCACAATCCCACTATCCTTCG)<br>PEX7-22 (AGGCGTGACTCGTAGAATGGACTG)	-	none	158

pex4-1 carries a C-to-T mutation at the 533rd nucleotide that destroys an *Mnl*I restriction enzyme cut site.

pex4-2 carries an intronic G-to-A mutation at the 1085th nucleotide (18 bp 5′ of the fourth exon of *PEX4*). We used the dCAPS website [http://helix.wustl.edu/dcaps/dcaps.html, 73] to design a genotyping marker for *pex4-2*.

pex5-1 carries a C-to-T mutation at the 2910th nucleotide that destroys an *Eco*RI restriction enzyme cut site [3].

pex5-10 has a T-DNA inserted in exon 5 and results the reduced and truncated pex5-10 protein [13].

The *35S:PEX5* construct expresses a *PEX5* cDNA [17]; for *35S:PEX5* genotyping, *PEX5* primers spanning introns were used to amplify larger genomic *PEX5* or the smaller *PEX5* cDNA products.

PEX7 lacks introns, so a primer from the *35S* promoter and a reverse *PEX7* primer were used to genotype *35S:PEX7* lines.
